# Does Pomegranate intake attenuate cardiovascular risk factors in hemodialysis patients?

**DOI:** 10.1186/1475-2891-13-18

**Published:** 2014-03-04

**Authors:** Lilach Shema-Didi, Batya Kristal, Shifra Sela, Ronit Geron, Liora Ore

**Affiliations:** 1 Quality Assurance Department, Western Galilee Hospital – Nahariya, Nahariya, Israel; 2 Nephrology Department, Western Galilee Hospital – Nahariya, Nahariya, Israel; 3 Faculty of Medicine in the Galilee, Bar Ilan University, Safed, Israel; 4 Clinical Microbiology Lab and Eliachar Research Lab, Western Galilee Hospital – Nahariya, Nahariya, Israel; 5 School of Public Health, Faculty of Social Welfare & Health Sciences, University of Haifa, Haifa, Israel

**Keywords:** Pomegranate juice hemodialysis, Polyphenols, Hypertension, Lipid profile

## Abstract

**Background:**

Atherosclerotic cardiovascular disease (CVD) is the most common cause of morbidity and mortality among hemodialysis (HD) patients. It has been attributed, among other causes, to hypertension and dyslipidemia. The aim of the present study was to investigate the effect of a year-long consumption of Pomegranate juice (PJ), on two traditional cardiovascular (CV) risk factors: hypertension and lipid profile, as well as on cardiovascular events.

**Methods:**

101 HD patients were randomized to receive 100 cc of PJ (0.7 mM polyphenols) or matching placebo juice, three times a week for one year. The primary endpoints were traditional CV risk factors; blood pressure and lipid profile. Systolic, diastolic and pulse pressure, plasma levels of triglycerides (TG), high density lipoprotein (HDL), low density lipoprotein (LDL) and total cholesterol were monitored quarterly during the study year. Secondary endpoint was incidence of cardiovascular events.

**Results:**

PJ consumption yielded a significant time response improvement in systolic blood pressure, pulse pressure, triglycerides and HDL level; an improvement that was not observed in the placebo intake group. These beneficial outcomes were more pronounced among patients with hypertension, high level of triglycerides and low levels of HDL.

**Conclusion:**

Regular PJ consumption by HD patients reduced systolic blood pressure and improved lipid profile. These favorable changes may reduce the accelerated atherosclerosis and high incidence of CVD among HD patients.

**Trial registration:**

ClinicalTrials.gov registry, Identifier number: NCT00727519

## Background

Patients on renal replacement therapy (RRT) are at increased risk of cardiovascular (CV) mortality and morbidity compared to the general population [1]. Every year, between 10-20% of all patients on dialysis die, with about 45% of deaths attributed to CV causes [2]. Established 'traditional’ atherosclerosis risk factors, such as hypertension and dyslipidemia, have been recognized as independent predictors of cardiovascular disease (CVD) among chronic kidney disease (CKD) [1] and hemodialysis (HD) patients [3,4]. Blood pressure is commonly high in HD patients. This phenomenon has been attributed to several causes, among them the chronic volume overload in HD patients, due to impaired blood pressure homoeostasis function [4]. In addition to the high prevalence of hypertension, HD patients usually display elevated triglycerides (TG), reduced high density lipoprotein (HDL) cholesterol and elevated concentration of lipoprotein-a [3,5], while total and low density lipoprotein (LDL) cholesterol usually remain within normal limits [5,6]. Several clinical trials and meta-analyses have shown the cardiovascular benefits of lowering blood pressure in patients with kidney disease [7,8] and patients on dialysis [4]. Although the cardiovascular benefits of improving lipid profile among dialysis patients is controversial [9], there is evidence that treatment of HD patients with lipid lowering drugs is associated with reduced CV mortality [10]. Therefore, improving lipid profile and reduction of blood pressure is a therapeutic target for patients on chronic dialysis.

It has been known for many years that high intake of fruits and vegetables is associated with reduced risk of coronary heart disease [11]. The beneficial effect of fruits and vegetables may be related especially to flavonoids, which are thought to exert their action by inhibiting LDL oxidation and platelet aggregation [12], as well as to inhibit the angiotensin converting enzyme (ACE), a key component in the renin angiotensin aldosterone system (RAAS) which regulates blood pressure [13]. Pomegranate juice (PJ) is a rich source of flavonoids and as such it has potent antioxidant activity. The flavonoids it contains have been linked to a diverse group of polyphenols, including ellagitanins, gallotannins and ellegic acid.

PJ antioxidant activity was studied mainly with regard to cardiovascular function among non HD patients. Different studies demonstrated the anti-atherogenicity properties of PJ by its ability to lower serum angiotensin converting enzyme (ACE) activity which resulted in systolic blood pressure reduction [13], decreased common carotid artery intima-media thickness (IMT) [14] and attenuation of the myocardial ischemia in patients who had congestive heart disease [15]. Recently, studies suggested that PJ consumption may be beneficial in populations at high risk to develop atherosclerosis and CVD [16,17]. The antioxidative effects of PJ were more impressive in diabetic patients than in healthy controls [17], leading to the assumption that PJ may have beneficial effect in patients exposed to oxidative stress (OS) burden. Since HD patients are exposed to the most severe systemic OS compared to other clinical states, PJ intake in this high risk population may be more effective than in other groups of patients. We have shown the beneficial effects of consistent consumption over one year's time of PJ on 'non traditional’ CV risk factors, such as OS and inflammation, and on clinical outcome such as reduction in intima media thickness [18]. Furthermore, we have demonstrated PJ's ability to reduce the incidence of infections, which is the second most common cause of morbidity and mortality of HD patients [18] However, the effect of PJ on 'traditional’ risk factors, such as hypertension and lipid profile has not yet been studied among HD patients.

The present study aims to characterize for the first time, the long term effects of PJ consumption by HD patients on hypertension, lipid profile and incidence of CVD.

## Methods

### Study population

One dialysis center at the Western Galilee Hospital, Nahariya, Israel participated in the study. Eligible participants were chronic HD patients aged >18 years who underwent 3 h HD sessions weekly using low-flux high performance cellulose-triacetate [sureflux- 190G, NIPRO] or polysulfone membranes [FX 10, Fresenius]. Exclusion criteria included: HD treatment for less than 3 months, simultaneous participation in other clinical trials, patient's refusal or inability to give informed consent due to mental or physical state, or pregnancy (actual or planned) during study period. One hundred and forty-nine HD patients were identified as eligible. Of these, 101 HD patients were recruited (detailed information regarding the study population was published in our previous paper [18]). Recruited patients did not differ from eligible subjects in demographic, dialysis and comorbidity characteristics [18]. All individuals gave their written informed consent to participate in the study. The study was approved by the Institutional Ethics Committee at the study center (Helsinki Committee approval no. 2008-06-06). The study was registered in ClinicalTrials.gov registration, Identifier number: NCT00727519.

### Study design

The study was a double-blind, placebo-controlled, randomized, clinical trial. Two groups of HD patients were compared: one group (n = 66) received 0.7 mmol of polyphenols in the form of 100 cc of PJ (Naturafood) and the other (n = 35) received a matching 100 cc placebo juice three times a week during the first dialysis treatment hour, for one year. Patients, medical and laboratory staff were all blinded to patient’s group allocation. The smaller randomization ratio of 2;1, with only a modest loss in statistical power [19] was chosen due to ethical (a potential PJ effect according to previous studies) and feasibility considerations. The study sample size (n = 101), with a ratio of 2:1, PJ: Placebo, was calculated to have 80% power (p = 0.05) to detect 5% decrease in SBP (as demonstrated previously among hypertensive patients [13]), assuming a mean SBP level of 140 ± 20 mm Hg according to our preliminary results.

During the study period patients were instructed not to drink any other fresh fruit juice at home. Verification of juice intake was carried out by the study investigator and was documented. Patients who did not drink the juice at least 3 times were excluded from the study. Blood for lipid profile measurements was drawn at 0, 3, 6 and 12 months of study intervention, always before dialysis, from the arterial sampling port closest to the patient. Three sequential blood pressure (BP) measurements performed before dialysis initiation were used to calculate the mean systolic and diastolic blood pressure (SBP, DBP) at 0, 3, 6 and 12 months of study intervention. Pulse pressure (PP) was calculated by SBP minus DBP.

#### PJ Juice

In order to choose the commercial PJ with the highest polyphenols levels, several hand squeezed and different commercial juices available in Israel were analyzed by the colorimetric assay in Migal Galilee Technology Center, as previously described [20,21]. PJ from Naturafood manufacturer [Turkey] was found to have the highest concentration of polyphenols, each 100 cc of PJ contained 0.7 mmol of polyphenols, and was used in this study. The juice was kept at room temperature <25°C until used.

#### Placebo Juice

The placebo was chosen to resemble the pomegranate juice in color and taste and was prepared especially for this study by food engineers. The placebo contained: pomegranate artificial extract of Frutarom Ltd; Citric acid; Caramel as color material; Aspartame and Acesulfame as sugar substitutes. Analyzing the placebo juice using colorimetric assay verified that the juice has no polyphenols. During placebo juice preparation, comparisons tests of flavor between placebo juice and Pomegranate Juice were performed by two independent testers to verify that placebo and Pomegranate Juice had a similar taste.

#### Outcomes

**The primary endpoint**s were traditional CV risk factors including: number of antihypertensive drugs taken at the end of follow up compared to study initiation, mean SBP, DBP and PP, as well as lipid profile (mean levels of TG, HDL, LDL, total cholesterol).

**Secondary outcome** was incidence of CVD. CVD events as a composite variable, consisted of hospitalizations due to acute myocardial infarction (MI) (fatal and non fatal); ischemic stroke; new events of peripheral vascular disease (excluding the arterio-venous fistula) and unstable angina. Non fatal MI was defined as the presence of at least two of the following criteria: chest pain of typical duration and intensity, and/or increased cardiac enzymes and diagnostic electrocardiogram changes. Fatal MI was defined as a death occurring within 24 h from hospital admission. Death occurring outside hospital for which no other cause was assigned was regarded as sudden death and was included in the definition of CVD event.

### Statistics

Data analysis was done with SPSS statistical analysis software. Continuous data are reported as mean ± SD. The t-test for independent samples, or Mann Whitney test when appropriate, were used to detect differences in continuous variables between treatment groups. Frequency counts were calculated for categorical data. Differences in these variables were assessed by Chi Square Tests. In cases when expected values were lower than necessary, Fisher Exact Test was used. In order to study the time effect of PJ/Placebo on hypertention and lipid profile, repeated measures and Bonferroni post hoc analyses were done separately in each of the groups. In order to retain the intention to treat analysis we used the last observation carried forward method for missing data.

Survival curve comparing the effect of treatment on CVD events was calculated by the Kaplan-Meier method using the intention-to-treat (ITT) principle.

All statistical tests were two-sided, and statistical significance was defined as P < 0.05.

## Results

### Characteristics of study population

Characteristics of the 101 patients are described in our previous paper [18]. Briefly, the two studied groups were similar at study initiation in demographics, co-morbidities, number of drugs and biochemical characteristics. The study population's mean age was 66.5 ± 11.8 years and 54.5% were males. Median follow-up time for the two groups was 12 months (range 0.25 – 12.6 months). The total dropout rate was 33.7%, insignificantly higher among PJ group (37.8%) as compared to the placebo group (25.7%). Patients who withdrew from the study did not differ from those who remained in their demographic, co-morbidy, treatment and biochemical characteristics. Furthermore, in spite of the 33.7% dropout rate, it should be noted that the baseline characteristics of patients who survived until the end of the study were similar at study initiation, indicating retained randomization success. Adverse events such as stomach upset or other GI-related effects were not demonstrated.

### Primary outcome

**Figure 1:**
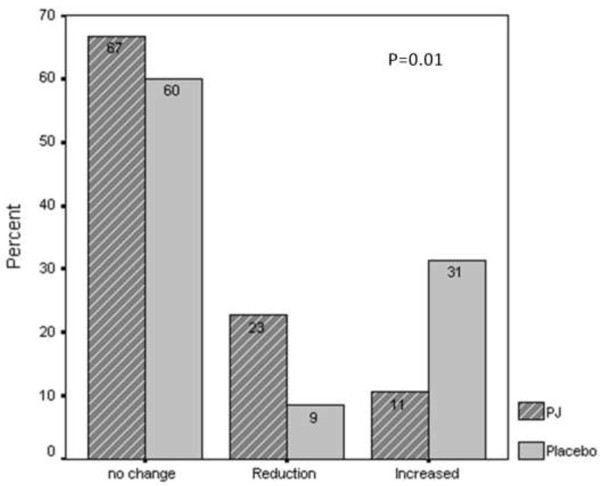
Changes in the number of anti HTN drugs after 12 months of intervention.

The distribution of the number of anti-hypertensive medications used at study entry was similar between the two studied groups (mean 1.5 ± 1.2 drugs among PJ compared to 1.1 ± 1.6 drugs among placebo group, p = 0.09). After one year of intervention a significant (P = 0.01) change in this distribution, as an ordinal variable (decrease, increase and no change) was noticed between the two group. The number of antihypertensive drugs decreased in 22.7% of the PJ patients, compared to 8.6% in the placebo group, while an increase was documented in 10.6% of the PJ patients compared to 31.4% in the placebo group (Figure 1). As shown in Table 1, among patients for whom the number of antihypertensive drugs did not change (66.7% (N = 44) and 60% (N = 21) of patients in the PJ and placebo group, respectively), treatment with PJ was associated with a significant time response reduction in SBP and PP. Mean SBP, among patients in the PJ group was reduced after one year of intervention by 6.8% (Mean delta 9.8 mm Hg, P.V of paired T-test = 0.01), while pulse pressure was reduced by 8.8% (Mean delta 6.6 mm Hg, P.V of paired T-test = 0.004). No significant changes were demonstrated among the placebo group. According to Bonferroni test, the SBP at 12 months' intervention was significantly lower than the level at study initiation and at 3 months' intervention.

**Table 1:** SBP, DBP and PP during the study period by treatment group, repeated measures and Bonferroni post hoc analysis, using last observation carried forward method for missing data

		**PJ**			**Placebo**			P^***** ^**for PJ vs. placebo at zero time and at 12 m**
	**Time**	**N**	**Mean ± SE (mm Hg)**	**P for trend**	**N**	**Mean ± SE (mm Hg)**	**P for trend**	
SBP^†^	0	44	145.6 ± 21.9	0.001	21	129.6 ± 27.2	0.15	0.28
	3 M		143.6 ± 23.9			131.2 ± 25.5		
	6 M		141.7 ± 20.4			138.8 ± 24.8		
	12 M		135.7 ± 21.3*			135.6 ± 27.7		0.96
SBP ≥ 140 at study initiation	0	26	157.8 ± 12.9	<0.001	5	162.0 ± 10.6	0.42	0.54
	3 M		153.2 ± 18.8			157.1 ± 13.9		
	6 M		149.5 ± 14.8**			156.2 ± 17.6		
	12 M		144.0 ± 16.9*			157.8 ± 10.3		0.12
DBP^††^	0	44	70.9 ± 11.4	0.09	21	62.0 ± 15.7	0.46	0.09
	3 M		70.6 ± 12.3			64.5 ± 13.5		
	6 M		69.6 ± 12.5			61.5 ± 16.5		
	12 M		67.7 ± 13.8			63.8 ± 20.4		0.38
PP^†††^	0	44	74.6 ± 19.5	0.04	21	64.3 ± 16.1	0.02	0.24
	3 M		72.9 ± 20.0			66.5 ± 19.2		
	6 M		72.1 ± 17.0			77.3 ± 14.5****		
	12 M		68.0 ± 16.6***			68.8 ± 14.2		0.84

*P < 0.05 for 12 m vs. 0 m and 3 m; **P < 0.05 for 6 m vs. zero time; ***P < 0.05 for 12 m vs. zero time; ****P < 0.05 for 6 m vs. zero time; *****T-test or Mann-whitny U test; †Systolic blood pressure; ††Diastolic blood pressure; ††† Pulse pressure.

Among patients with baseline SBP ≥140 mm Hg (N = 26 for PJ group, N = 5 for placebo group) the significant effect of PJ consumption on blood pressure reduction occurred earlier (after 6 m of intervention) and was more pronounced: SBP was reduced by 5.3% after 6 m, and by 8.7% after 12 m of intervention (Table 1). No changes were observed in DBP in both groups.

Among patients in the placebo group a significantly increased PP was demonstrated. PJ consumption not only attenuated this increase but also significantly decreased it (Table 1).

Normal values of LDL and total cholesterol were demonstrated for the two study groups (Table 2). Treatment with PJ was not associated with significant changes in the above parameters. However, it was associated with significant time response improvements in HDL and TG levels, more evidently among patients with a pathologic level of these parameters (Table 2). Among patients with abnormal levels, following 12 months of intervention, levels of HDL were significantly higher while levels of TG significantly decreased among PJ patients as compared to the placebo subjects (Table 2).

**Table 2:** Lipid profile during the study period by treatment group, repeated measures and Bonferroni post hoc analysis, using last observation carried forward method for missing data

		**PJ**			**Placebo**			P^*** ^**for PJ vs. placebo at 12 m**
		**N**	**Mean ± SD**	**P for trend**	**N**	**Mean ± SD**	**P for trend**	
LDL mg/dl	0	66	95.1 ± 31.5	0.07	35	95.8 ± 28.9	0.38	0.85
	3 M		98.9 ± 35.6			98.0 ± 28.1		
	6 M		95.4 ± 32.4			91.8 ± 25.1		
	12 M		100.0 ± 33.1			94.3 ± 27.2		0.39
Total cholesterol mg/dl	0	66	164.8 ± 37.1	0.27	35	163.5 ± 33.4	0.18	0.86
	3 M		167.7 ± 40.7			171.7 ± 35.1		
	6 M		163.4 ± 38.9			164.8 ± 38.0		
	12 M		167.3 ± 43.5			165.1 ± 35.8		0.79
HDL mg/dl	0	66	33.1 ± 9.5*	<0.001	35	36.6 ± 10.7	0.18	0.10
	3 M		37.2 ± 11.3			37.0 ± 11.7		
	6 M		36.6 ± 10.9			37.3 ± 12.9		
	12 M		36.8 ± 10.8			34.3 ± 15.4		0.40
HDL ≤40 mg/dl at study initiation	0	54	29.7 ± 5.7*	<0.001	23	30.2 ± 5.6	0.09	0.84
	3 M		33.8 ± 7.8			31.2 ± 7.6		
	6 M		33.6 ± 7.7			31.8 ± 10.2		
	12 M		33.6 ± 7.5			27.6 ± 11.6		0.03
TG mg/dl	0	66	183.6 ± 101.6**	0.05	35	176.7 ± 86.7	0.04	0.73
	3 M		175.7 ± 90.8			192.9 ± 100.7		
	6 M		174.0 ± 89.8			193.6 ± 90.5		
	12 M		167.3 ± 86.3			206.1 ± 109.4		0.05
TG ≥200 mg/dl at study initiation	0	20	310.1 ± 87.8**	0.02	10	288.1 ± 71.9	0.15	0.13
	3 M		267.3 ± 86.6			318.2 ± 96.6		
	6 M		248.7 ± 100.5			301.5 ± 78.2		
	12 M		237.4 ± 102.5			320.4 ± 56.8		0.008

*P < 0.05 for zero time vs. 3, 6 and 12 m; **P < 0.05 for zero time vs. 6 m and 12 m; ***T-test or Mann-whitny U test.

### Secondary outcomes

#### CVD events

**Figure 2:**
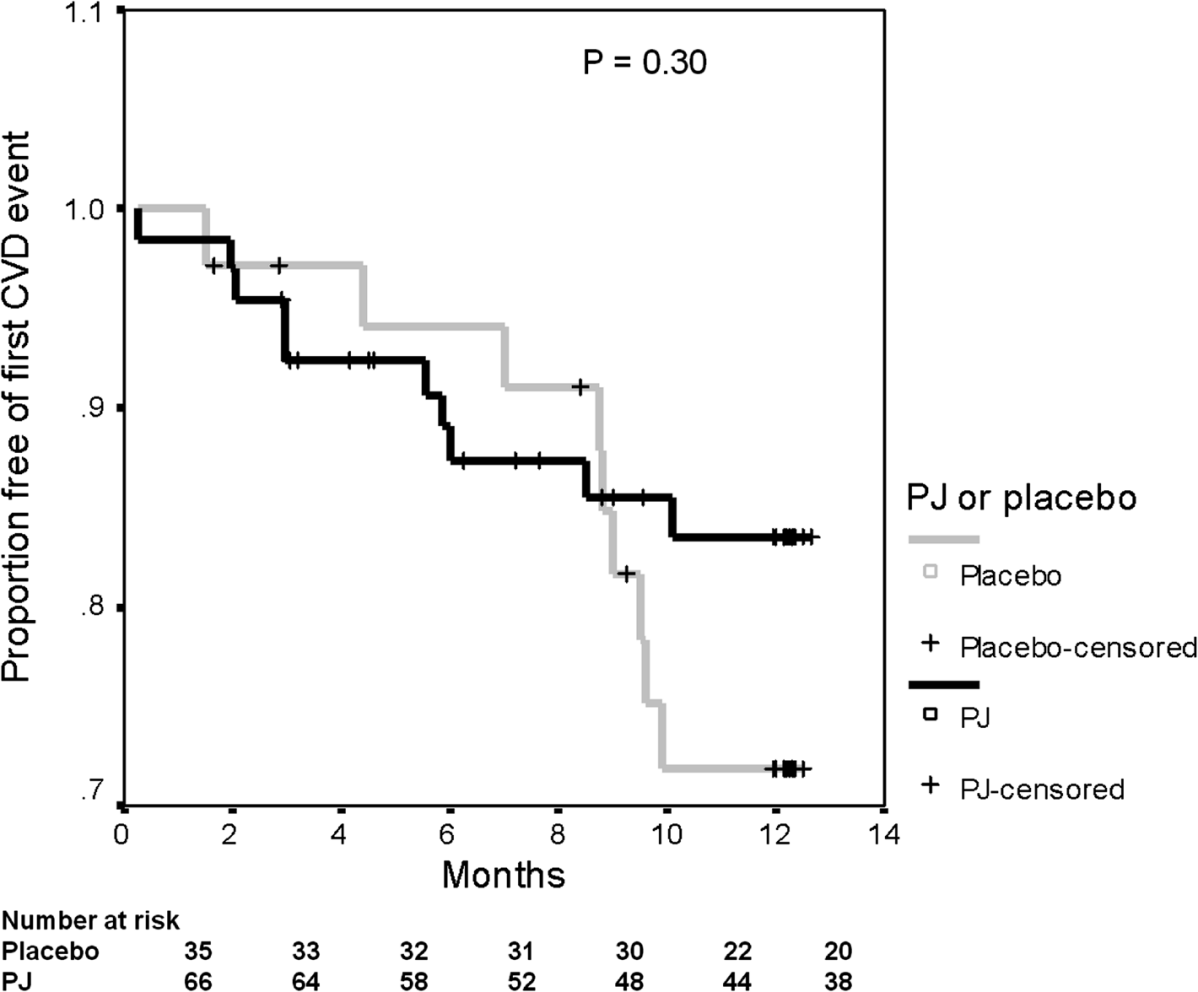
Kaplan-Meier survival curves following first CVD event.

The CVD incidence rates by group allocation, rate ratios and attributable risks are shown in Table 3. As seen, PJ intake, as compared to Placebo intake, was associated with 39% and 46% incidence rate reduction of the first and second CVD events, respectively. In addition, the reduction of the first event, as compared to the second event, was more pronounced in the PJ group (5.4 times lower incidence rate), as compared to the placebo group (4.7 times lower rate). Due to a small number of events, the difference between the two groups, as expressed by the Kaplan-Meier survival analysis, did not reach statistical significance (Figure 2).

**Table 3:** Effect of PJ intake on the incidence rate, rate ratio and attributable risk of first and second CVD events

**CV Events**	**Group**	**Incidence rate**	**Rate ratio**	**P.V (95% CI)**	**Attribute risk**
First event	PJ	10648.24PM×1000=15.421000PM	0.61	0.30 (0.25-1.5)	25.031000PM-15.421000PM25.031000PM×100=38.4%
	Placebo	9359.57PM×1000=25.031000PM			
Second event	PJ	2701.42PM×1000=2.851000PM	0.54	0.55 (0.08-3.9)	5.341000PM-2.851000PM5.341000PM×100=46.3%
	Placebo	2376.46PM×1000=5.311000PM			

## Discussion

In the present study we demonstrated that intake of polyphenols-rich commercial PJ with anti-atherogenicity properties [13], improved the levels of SBP, PP, TG, and HDL among HD patients. These effects were more notable among patients with higher levels of SBP and TG and among subjects with lower levels of HDL. PJ intake was also found to be related to the decrease in the number of anti hypertensive drugs, highlighting its beneficial effect on blood pressure level. It must be emphasized that when comparing the two treatment groups HDL and TG were the only parameters who demonstrated significant differences, which might be explained for both factors by an improvement in the PJ group and impairment in the placebo group. Furthermore, PJ had no demonstrable effect on total cholesterol and LDL levels, probably due to their normal levels at study entry. Our results are in agreement with studies conducted in other chronic populations, demonstrating reduction in SBP [13] and improvement in lipid profiles among patients who consumed PJ [16]. Our study demonstrated for the first time the anti-hypertensive and anti- hyperlipidemic properties of PJ among HD patients.

It is widely accepted that hypertension and dyslipidemia are contributors to the atherosclerotic process, as well as independent predictors of CVD in CKD and dialysis patients [1]. As such, our results in the current and previous study [18] indicate the potentially important cardioprotective role of PJ. In spite of the above we did not succeed to show a significant reduction in the incidence of CVD, probably due to the longer intervention period needed to demonstrate a statistically significant effect.

The mechanism by which PJ consumption reduced SBP may relate to its ability to decrease ACE activity (secondary to its antioxidant properties) or may be due to a direct effect on serum ACE activity [13]. Furthermore, as reactive oxygen species (ROS) contribute to endothelium dependent contraction and to increased vascular resistance, the antioxidative effects by PJ, as we previously demonstrated [18], can possibly restore endothelial function and hence decrease blood pressure [13,22]. SBP reduction may also be a result of PJ's ability to protect nitric oxide against oxidative destruction [23] and to enhance the nitric oxide synthase bioactivity [24]. In contrast to SBP reduction, the mechanism underlying the anti- hyperlipidemic effect of PJ is unclear. Both an inhibition of intestinal absorption, as well as accelerated clearance of plasma triglycerides lipid may account for the observed hypolipemic action of PJ [25]. The beneficial effects of PJ on HDL and TGs could be mediated by its ability to induce increment in paraoxonase 1 [26], protecting HDL from oxidation [27,28], and in paraoxonase 2 [29], influencing TG accumulation by macrophages as well their TG synthesis [30].

Important questions related to interpretation of our above results are whether the associations found are real and causal. Taking into consideration the uncompromising methodology described, there is no reason to believe that biases have been introduced. As for causality, all Hill’s criteria were met: the clinical trial methodology assured temporal relationship issues; the protective associations between PJ exposure and cardiovascular events were found strong; our findings regarding the association between polyphenols- rich juice and its anti-hypertensive and hypolipemic properties were consistently described across other studies of divergent designs and populations [13,16,25]; a time response relationship was demonstrated between the intervention duration and outcomes; and there is a possible biological explanation for the relationships noticed between PJ intake and the demonstrated results (described above). Nevertheless, some methodological limitations should be mentioned: first, this study was a small single center trial, which may limit its external validity (generalizability); though the sample size was adequate to examine all study assumptions. In addition, data collection by one investigator in one trial center assured high quality of data collected. Second, there is no data regarding the pharmacokinetics of polyphenols gastrointestinal absorption among HD patients. However, the effect on SBP and lipid profile was demonstrated only among the PJ group, supporting the notion of a PJ effect.

## Conclusions

The significant beneficial effect of PJ intake on SBP, PP and lipid profile among HD patients, in addition to its beneficial effect on oxidative stress and inflammation, suggests that constant PJ consumption can offer wide protection against cardiovascular events - the main cause of morbidity and mortality among HD patients.

Furthermore, as controlled consumption of PJ has been shown to lower morbidities in HD patients it is expected to reduce costs associated with those patients' care. Further multi centered clinical studies are needed to substantiate our findings that while directly improving patients' quality of life, PJ can also significantly reduce health expenditure. Such studies might well influence future policy makers to include PJ as part of state-funded standard care for HD patients.

## Abbreviations

CVD: Cardiovascular disease; HD: Hemodialysis; PJ: Pomegranate juice; CV: Cardiovascular; TG: Triglycerides; HDL: High density lipoprotein; LDL: Low density lipoprotein; RRT: Renal replacement therapy; CKD: Chronic kidney disease; ACE: Angiotensin converting enzyme; RAAS: Renin angiotensin aldosterone system; IMT: Intima-media thickness; BP: Blood pressure; SBP: Systolic blood pressure; DBP: Diastolic blood pressure; PP: Pulse pressure; MI: Myocardial infarction; ITT: Intention-to-treat.

## Competing interests

The authors declare that they have no competing interests.

## Authors’ contributions

All authors have contributed to the conception and design of the work. We had full access to all of the data in this study, and we take complete responsibility for the integrity of the data and the accuracy of the data analysis. We declare that we have seen and approved the final version.
